# (2,2′-Bipyridine-κ^2^ *N*,*N*′)bis­(4-methyl­benzoato-κ^2^ *O*,*O*′)lead(II)

**DOI:** 10.1107/S1600536810005544

**Published:** 2010-02-13

**Authors:** Jun Dai, Juan Yang, Yingjie Li

**Affiliations:** a Institute of Safety Science and Engineering, Henan Polytechnic University, Jiaozuo, 454003, People’s Republic of China; b Department of Physical Chemistry, Henan Polytechnic University, Jiaozuo, 454003, People’s Republic of China

## Abstract

In the title compound, [Pb(C_8_H_7_O_2_)_2_(C_10_H_8_N_2_)], the Pb^II^ ion is coordinated by two N atoms from one 2,2′-bipyridine ligand and four O atoms from two 4-methyl­benzoate anions in a distorted pseudo-square-pyramidal environment, considering one of the carboxyl­ate anions as an apical ligand. Pairs of complex mol­ecules related by inversion centers are organized into dimers *via* pairs of Pb⋯O inter­actions [3.185 (2) Å] and stacking interactions between 2,2′-bipyridine and 4-methyl­benzoate ligands, with a mean distance between their planes of 3.491 Å.

## Related literature

For potential applications of lead compounds, see: Fan & Zhu (2006 ▶); Hamilton *et al.* (2004 ▶). For the use of aromatic carboxyl­ates and 2,2′-bipyridine-type ligands in the preparation of metal complexes, see: Wang *et al.* (2006 ▶); Masaoka *et al.* (2001 ▶).
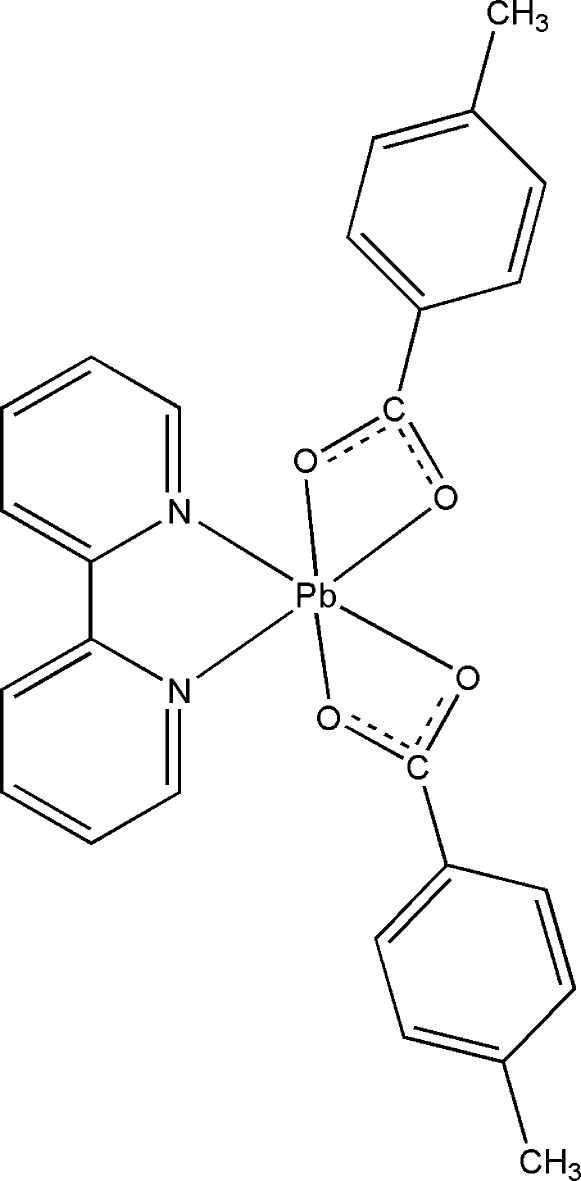


## Experimental

### 

#### Crystal data

[Pb(C_8_H_7_O_2_)_2_(C_10_H_8_N_2_)]
*M* *_r_* = 633.65
Triclinic, 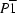

*a* = 9.5510 (11) Å
*b* = 10.0805 (12) Å
*c* = 13.2483 (15) Å
α = 109.865 (1)°
β = 97.322 (1)°
γ = 90.643 (1)°
*V* = 1187.8 (2) Å^3^
*Z* = 2
Mo *K*α radiation
μ = 7.14 mm^−1^
*T* = 296 K
0.35 × 0.26 × 0.18 mm

#### Data collection

Bruker APEXII CCD area detector diffractometer
Absorption correction: multi-scan (*SADABS*; Bruker, 2007 ▶) *T*_min_ = 0.124, *T*_max_ = 0.277
14285 measured reflections
5555 independent reflections
4965 reflections with *I* > 2σ(*I*)
*R*_int_ = 0.027

#### Refinement

*R*[*F* ^2^ > 2σ(*F* ^2^)] = 0.023
*wR*(*F* ^2^) = 0.051
*S* = 1.02
5555 reflections
300 parameters
2 restraints
H-atom parameters constrained
Δρ_max_ = 0.77 e Å^−3^
Δρ_min_ = −0.76 e Å^−3^

### 

Data collection: *APEX2* (Bruker, 2007 ▶); cell refinement: *SAINT* (Bruker, 2007 ▶); data reduction: *SAINT*; program(s) used to solve structure: *SHELXS97* (Sheldrick, 2008 ▶); program(s) used to refine structure: *SHELXL97* (Sheldrick, 2008 ▶); molecular graphics: *SHELXTL* (Sheldrick, 2008 ▶); software used to prepare material for publication: *SHELXTL*.

## Supplementary Material

Crystal structure: contains datablocks global, I. DOI: 10.1107/S1600536810005544/gk2256sup1.cif

Structure factors: contains datablocks I. DOI: 10.1107/S1600536810005544/gk2256Isup2.hkl

Additional supplementary materials:  crystallographic information; 3D view; checkCIF report

## Figures and Tables

**Table 1:** Selected bond lengths (Å)

Pb1—O1	2.333 (2)
Pb1—O3	2.418 (2)
Pb1—N2	2.608 (3)
Pb1—O2	2.644 (2)
Pb1—N1	2.656 (3)
Pb1—O4	2.701 (2)

**Fig. 1.**
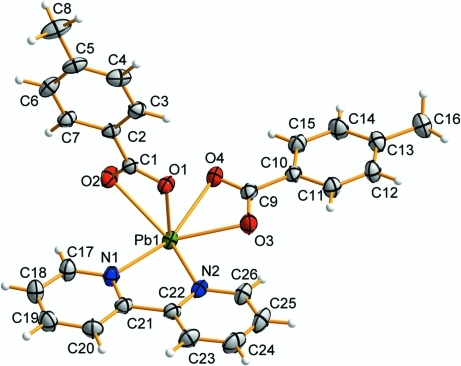
The coordination environment around PbII in the title complex with the atom-labeling scheme. Displacement ellipsoids for non-hydrogen atoms are drawn at the 30% probability level.

## supplementary crystallographic information

### Comment

Recently, lead compounds have been increasingly studied owing to their possible applications in different fields (Fan *et al.*, 2006; Hamilton *et al.*, 2004), such as ion-exchange, nonlinear optics and catalysis, especially in environmental protection due to the toxicity of lead and in biological systems for its diverse interactions with biological molecules. As an important family of multidentate O-donor ligands, aromatic carboxylate ligands have been extensively employed in the preparation of metal-organic complexes because of their potential properties and intriguing structural topologies (Wang *et al.*, 2006; Masaoka *et al.*, 2001).  Herein, we report the structure of the title complex.

The asymmetric unit of the title complex, [Pb(C_8_H_7_O_2_)_2_(C_10_H_8_N_2_)], contains a Pb^II^ cation, two 4-methylbenzoate ligands, one 2,2'-bipyridine ligand, as illustrated in Fig. 1. The Pb^II^ atom is hexacoordinated being chelated by two carboxylate groups and one 2,2'-bipyridine. The Pb—O bond lengths are in the range of 2.333 (2) to 2.701 (2) Å. The Pb—N bond lengths are 2.608 (3) to 2.656 (3) Å. The Pb^II^ atom has a distorted pseudo-square-pyramidal environment, considering one of the carboxylate anions as an apical ligand. The complex molecules related by inversion center are organized into dimeric units via a pair of Pb···O interactions of 3.185 (2) Å and stacking inteactions between 2,2'-bipyridine and 4-methylbenzoate ligands, with a mean distance between their planes of 3.491 Å.

### Experimental

A mixture of Pb(CH_3_COO)_2_^.^3H_2_O (0.199 g, 0.52 mmol), 4-methylbenzoic acid (0.114 g, 0.84 mmol), 2,2'-bipyridine (0.033 g, 0.21 mmol) and distilled water (10 ml) was sealed in a 25 ml Teflon-lined stainless autoclave. The mixture was heated at 403 K for 7 days to give the colorless crystals suitable for X-ray diffraction analysis.

### Refinement

All H atoms bounded to C atoms were placed in calculated positions and treated in a riding-model approximation, with C (aromatic)—H = 0.93 Å, *C*(methyl)—H = 0.96 Å and *U*_iso_(H_aromatic_) = 1.2U_eq_(C), *U*_iso_(H_methyl_) = 1.5U_eq_(C). Two rigid-bond restraints to Uij values (DELU) were imposed on bonded atoms Pb1—O4 and Pb1—O2.

### Crystal data

**Table:**

[Pb(C_8_H_7_O_2_)_2_(C_10_H_8_N_2_)]	*Z* = 2
*M**_r_* = 633.65	*F*(000) = 612
Triclinic, *P*1	*D*_x_ = 1.772 Mg m^−^^3^
Hall symbol: -P 1	Mo *K*α radiation, λ = 0.71073 Å
*a* = 9.5510 (11) Å	Cell parameters from 7017 reflections
*b* = 10.0805 (12) Å	θ = 2.2–26.6°
*c* = 13.2483 (15) Å	µ = 7.14 mm^−^^1^
α = 109.865 (1)°	*T* = 296 K
β = 97.322 (1)°	Prism, colorless
γ = 90.643 (1)°	0.35 × 0.26 × 0.18 mm
*V* = 1187.8 (2) Å^3^	

### Data collection

**Table:**

Bruker APEXII CCD area detector diffractometer	5555 independent reflections
Radiation source: fine-focus sealed tube	4965 reflections with *I* > 2σ(*I*)
graphite	*R*_int_ = 0.027
φ and ω scans	θ_max_ = 27.8°, θ_min_ = 2.2°
Absorption correction: multi-scan (*SADABS*; Bruker, 2007)	*h* = −12→12
*T*_min_ = 0.124, *T*_max_ = 0.277	*k* = −13→13
14285 measured reflections	*l* = −17→17

### Refinement

**Table:**

Refinement on *F*^2^	Primary atom site location: structure-invariant direct methods
Least-squares matrix: full	Secondary atom site location: difference Fourier map
*R*[*F*^2^ > 2σ(*F*^2^)] = 0.023	Hydrogen site location: inferred from neighbouring sites
*wR*(*F*^2^) = 0.051	H-atom parameters constrained
*S* = 1.02	*w* = 1/[σ^2^(*F*_o_^2^) + (0.0227*P*)^2^ + 0.140*P*] where *P* = (*F*_o_^2^ + 2*F*_c_^2^)/3
5555 reflections	(Δ/σ)_max_ = 0.001
300 parameters	Δρ_max_ = 0.77 e Å^−^^3^
2 restraints	Δρ_min_ = −0.76 e Å^−^^3^

### Fractional atomic coordinates and isotropic or equivalent isotropic displacement parameters (Å^2^)

**Table:**

	*x*	*y*	*z*	*U*_iso_*/*U*_eq_	
Pb1	0.010726 (12)	0.117736 (12)	0.677133 (9)	0.03988 (5)	
O1	0.2108 (2)	0.1792 (2)	0.80800 (18)	0.0483 (5)	
O2	0.0648 (2)	0.3523 (2)	0.84728 (18)	0.0543 (6)	
O3	0.1789 (3)	0.0320 (3)	0.5511 (2)	0.0601 (6)	
O4	0.1595 (3)	0.2603 (2)	0.58236 (19)	0.0556 (6)	
N1	−0.0850 (3)	0.0481 (3)	0.8332 (2)	0.0468 (6)	
N2	0.0489 (3)	−0.1362 (3)	0.6814 (2)	0.0454 (6)	
C1	0.1748 (3)	0.2982 (3)	0.8699 (3)	0.0413 (7)	
C2	0.2682 (3)	0.3698 (3)	0.9753 (3)	0.0409 (7)	
C3	0.4063 (3)	0.3339 (3)	0.9934 (3)	0.0488 (8)	
H3A	0.4430	0.2632	0.9395	0.059*	
C4	0.4895 (4)	0.4025 (4)	1.0911 (3)	0.0586 (10)	
H4A	0.5825	0.3781	1.1016	0.070*	
C5	0.4386 (4)	0.5062 (4)	1.1735 (3)	0.0559 (9)	
C6	0.3003 (4)	0.5395 (4)	1.1556 (3)	0.0576 (9)	
H6A	0.2630	0.6076	1.2108	0.069*	
C7	0.2156 (4)	0.4740 (3)	1.0576 (3)	0.0470 (7)	
H7A	0.1233	0.5000	1.0470	0.056*	
C8	0.5337 (5)	0.5824 (5)	1.2781 (4)	0.0861 (15)	
H8A	0.5932	0.5164	1.2977	0.129*	
H8B	0.4770	0.6244	1.3343	0.129*	
H8C	0.5913	0.6548	1.2687	0.129*	
C9	0.2085 (3)	0.1461 (4)	0.5346 (3)	0.0449 (7)	
C10	0.3033 (3)	0.1363 (3)	0.4516 (2)	0.0413 (7)	
C11	0.3697 (4)	0.0135 (4)	0.4040 (3)	0.0522 (8)	
H11A	0.3569	−0.0640	0.4254	0.063*	
C12	0.4548 (4)	0.0043 (4)	0.3251 (3)	0.0571 (9)	
H12A	0.4985	−0.0792	0.2947	0.068*	
C13	0.4757 (4)	0.1168 (4)	0.2909 (3)	0.0532 (8)	
C14	0.4090 (4)	0.2395 (4)	0.3393 (3)	0.0662 (11)	
H14A	0.4209	0.3168	0.3173	0.079*	
C15	0.3258 (4)	0.2498 (4)	0.4188 (3)	0.0584 (9)	
H15A	0.2844	0.3342	0.4508	0.070*	
C16	0.5663 (5)	0.1044 (5)	0.2032 (3)	0.0709 (11)	
H16A	0.6455	0.0495	0.2118	0.106*	
H16B	0.5998	0.1970	0.2084	0.106*	
H16C	0.5113	0.0589	0.1335	0.106*	
C17	−0.1533 (4)	0.1412 (4)	0.9053 (3)	0.0553 (9)	
H17A	−0.1560	0.2331	0.9046	0.066*	
C18	−0.2200 (4)	0.1079 (4)	0.9806 (3)	0.0599 (10)	
H18A	−0.2657	0.1759	1.0301	0.072*	
C19	−0.2174 (4)	−0.0266 (4)	0.9810 (3)	0.0640 (10)	
H19A	−0.2606	−0.0521	1.0313	0.077*	
C20	−0.1495 (4)	−0.1250 (4)	0.9053 (3)	0.0600 (10)	
H20A	−0.1487	−0.2182	0.9032	0.072*	
C21	−0.0830 (3)	−0.0847 (3)	0.8330 (3)	0.0435 (7)	
C22	−0.0052 (3)	−0.1848 (3)	0.7519 (3)	0.0441 (7)	
C23	0.0131 (5)	−0.3217 (4)	0.7493 (4)	0.0672 (11)	
H23A	−0.0239	−0.3531	0.7993	0.081*	
C24	0.0856 (5)	−0.4114 (4)	0.6734 (4)	0.0755 (12)	
H24A	0.0987	−0.5035	0.6713	0.091*	
C25	0.1376 (5)	−0.3618 (4)	0.6014 (4)	0.0736 (12)	
H25A	0.1864	−0.4203	0.5485	0.088*	
C26	0.1179 (4)	−0.2249 (4)	0.6073 (3)	0.0600 (9)	
H26A	0.1542	−0.1925	0.5575	0.072*	

### Atomic displacement parameters (Å^2^)

**Table:**

	*U*^11^	*U*^22^	*U*^33^	*U*^12^	*U*^13^	*U*^23^
Pb1	0.04568 (8)	0.03734 (7)	0.03350 (7)	0.00681 (5)	0.00661 (5)	0.00766 (5)
O1	0.0490 (13)	0.0417 (13)	0.0455 (12)	0.0100 (10)	0.0040 (10)	0.0047 (10)
O2	0.0543 (14)	0.0485 (12)	0.0465 (12)	0.0145 (11)	−0.0036 (11)	0.0024 (8)
O3	0.0754 (17)	0.0499 (15)	0.0603 (15)	0.0102 (12)	0.0314 (13)	0.0178 (12)
O4	0.0650 (15)	0.0465 (14)	0.0517 (14)	0.0039 (11)	0.0230 (11)	0.0069 (11)
N1	0.0555 (17)	0.0416 (15)	0.0474 (16)	0.0116 (12)	0.0152 (13)	0.0175 (13)
N2	0.0493 (16)	0.0372 (14)	0.0425 (15)	0.0101 (12)	0.0068 (12)	0.0041 (12)
C1	0.0449 (18)	0.0384 (17)	0.0400 (16)	0.0004 (13)	0.0069 (13)	0.0122 (14)
C2	0.0449 (17)	0.0317 (16)	0.0451 (17)	−0.0002 (13)	0.0026 (13)	0.0132 (13)
C3	0.0450 (18)	0.0385 (18)	0.062 (2)	0.0048 (14)	0.0081 (16)	0.0162 (16)
C4	0.047 (2)	0.045 (2)	0.082 (3)	−0.0008 (16)	−0.0102 (18)	0.025 (2)
C5	0.067 (2)	0.0364 (18)	0.059 (2)	−0.0093 (16)	−0.0156 (18)	0.0191 (17)
C6	0.076 (3)	0.0364 (18)	0.051 (2)	0.0046 (17)	0.0001 (18)	0.0063 (15)
C7	0.0463 (18)	0.0387 (18)	0.0507 (19)	0.0068 (14)	0.0017 (15)	0.0102 (15)
C8	0.100 (4)	0.059 (3)	0.082 (3)	−0.013 (2)	−0.036 (3)	0.019 (2)
C9	0.0417 (17)	0.0466 (19)	0.0370 (16)	0.0020 (14)	0.0028 (13)	0.0035 (14)
C10	0.0392 (16)	0.0446 (18)	0.0352 (15)	0.0013 (13)	0.0022 (12)	0.0084 (13)
C11	0.056 (2)	0.050 (2)	0.055 (2)	0.0112 (16)	0.0145 (16)	0.0217 (17)
C12	0.059 (2)	0.056 (2)	0.059 (2)	0.0196 (17)	0.0222 (18)	0.0187 (19)
C13	0.0469 (19)	0.066 (2)	0.050 (2)	0.0074 (17)	0.0112 (15)	0.0227 (18)
C14	0.076 (3)	0.059 (2)	0.075 (3)	0.009 (2)	0.031 (2)	0.031 (2)
C15	0.066 (2)	0.045 (2)	0.063 (2)	0.0092 (17)	0.0198 (19)	0.0133 (18)
C16	0.065 (2)	0.090 (3)	0.068 (3)	0.015 (2)	0.028 (2)	0.033 (2)
C17	0.068 (2)	0.050 (2)	0.053 (2)	0.0179 (17)	0.0216 (18)	0.0189 (17)
C18	0.066 (2)	0.066 (3)	0.052 (2)	0.0174 (19)	0.0237 (18)	0.0201 (19)
C19	0.066 (2)	0.070 (3)	0.064 (2)	0.004 (2)	0.023 (2)	0.029 (2)
C20	0.071 (2)	0.048 (2)	0.068 (2)	0.0028 (18)	0.018 (2)	0.0268 (19)
C21	0.0435 (18)	0.0357 (17)	0.0495 (18)	0.0037 (13)	0.0053 (14)	0.0129 (14)
C22	0.0453 (18)	0.0346 (17)	0.0486 (18)	0.0016 (13)	0.0036 (14)	0.0105 (14)
C23	0.080 (3)	0.040 (2)	0.082 (3)	0.0076 (19)	0.016 (2)	0.019 (2)
C24	0.089 (3)	0.036 (2)	0.093 (3)	0.015 (2)	0.010 (3)	0.011 (2)
C25	0.087 (3)	0.050 (2)	0.071 (3)	0.030 (2)	0.018 (2)	0.001 (2)
C26	0.069 (2)	0.048 (2)	0.057 (2)	0.0158 (18)	0.0149 (18)	0.0071 (17)

### Geometric parameters (Å, °)

**Table:**

Pb1—O1	2.333 (2)	C10—C11	1.386 (4)
Pb1—O3	2.418 (2)	C11—C12	1.383 (5)
Pb1—N2	2.608 (3)	C11—H11A	0.9300
Pb1—O2	2.644 (2)	C12—C13	1.378 (5)
Pb1—N1	2.656 (3)	C12—H12A	0.9300
Pb1—O4	2.701 (2)	C13—C14	1.389 (5)
O1—C1	1.282 (4)	C13—C16	1.509 (5)
O2—C1	1.239 (4)	C14—C15	1.376 (5)
O3—C9	1.277 (4)	C14—H14A	0.9300
O4—C9	1.240 (4)	C15—H15A	0.9300
N1—C17	1.335 (4)	C16—H16A	0.9600
N1—C21	1.339 (4)	C16—H16B	0.9600
N2—C26	1.333 (4)	C16—H16C	0.9600
N2—C22	1.345 (4)	C17—C18	1.378 (5)
C1—C2	1.500 (4)	C17—H17A	0.9300
C2—C7	1.384 (4)	C18—C19	1.358 (5)
C2—C3	1.385 (4)	C18—H18A	0.9300
C3—C4	1.379 (5)	C19—C20	1.384 (5)
C3—H3A	0.9300	C19—H19A	0.9300
C4—C5	1.375 (5)	C20—C21	1.378 (5)
C4—H4A	0.9300	C20—H20A	0.9300
C5—C6	1.377 (5)	C21—C22	1.486 (5)
C5—C8	1.508 (5)	C22—C23	1.382 (5)
C6—C7	1.383 (5)	C23—C24	1.372 (6)
C6—H6A	0.9300	C23—H23A	0.9300
C7—H7A	0.9300	C24—C25	1.359 (6)
C8—H8A	0.9600	C24—H24A	0.9300
C8—H8B	0.9600	C25—C26	1.372 (5)
C8—H8C	0.9600	C25—H25A	0.9300
C9—C10	1.490 (4)	C26—H26A	0.9300
C10—C15	1.379 (5)		
			
O1—Pb1—O3	84.31 (9)	C15—C10—C11	118.0 (3)
O1—Pb1—N2	83.74 (8)	C15—C10—C9	120.4 (3)
O3—Pb1—N2	77.33 (8)	C11—C10—C9	121.6 (3)
O1—Pb1—O2	52.25 (7)	C12—C11—C10	121.2 (3)
O3—Pb1—O2	121.72 (8)	C12—C11—H11A	119.4
N2—Pb1—O2	124.68 (8)	C10—C11—H11A	119.4
O1—Pb1—N1	79.77 (8)	C13—C12—C11	121.0 (3)
O3—Pb1—N1	137.27 (8)	C13—C12—H12A	119.5
N2—Pb1—N1	61.75 (8)	C11—C12—H12A	119.5
O2—Pb1—N1	77.17 (8)	C12—C13—C14	117.4 (3)
O1—Pb1—O4	82.72 (8)	C12—C13—C16	120.5 (3)
O3—Pb1—O4	50.77 (8)	C14—C13—C16	122.1 (3)
N2—Pb1—O4	127.29 (8)	C15—C14—C13	121.8 (4)
O2—Pb1—O4	83.26 (7)	C15—C14—H14A	119.1
N1—Pb1—O4	159.20 (9)	C13—C14—H14A	119.1
C1—O1—Pb1	99.27 (19)	C14—C15—C10	120.6 (3)
C1—O2—Pb1	85.80 (18)	C14—C15—H15A	119.7
C9—O3—Pb1	99.36 (19)	C10—C15—H15A	119.7
C9—O4—Pb1	87.0 (2)	C13—C16—H16A	109.5
C17—N1—C21	118.3 (3)	C13—C16—H16B	109.5
C17—N1—Pb1	119.9 (2)	H16A—C16—H16B	109.5
C21—N1—Pb1	121.1 (2)	C13—C16—H16C	109.5
C26—N2—C22	118.0 (3)	H16A—C16—H16C	109.5
C26—N2—Pb1	119.3 (2)	H16B—C16—H16C	109.5
C22—N2—Pb1	122.6 (2)	N1—C17—C18	123.3 (3)
O2—C1—O1	122.5 (3)	N1—C17—H17A	118.4
O2—C1—C2	119.8 (3)	C18—C17—H17A	118.4
O1—C1—C2	117.7 (3)	C19—C18—C17	118.5 (4)
C7—C2—C3	118.6 (3)	C19—C18—H18A	120.7
C7—C2—C1	119.6 (3)	C17—C18—H18A	120.7
C3—C2—C1	121.8 (3)	C18—C19—C20	118.9 (3)
C4—C3—C2	120.3 (3)	C18—C19—H19A	120.5
C4—C3—H3A	119.9	C20—C19—H19A	120.5
C2—C3—H3A	119.9	C21—C20—C19	119.8 (3)
C5—C4—C3	121.7 (3)	C21—C20—H20A	120.1
C5—C4—H4A	119.2	C19—C20—H20A	120.1
C3—C4—H4A	119.2	N1—C21—C20	121.1 (3)
C4—C5—C6	117.7 (3)	N1—C21—C22	116.7 (3)
C4—C5—C8	120.4 (4)	C20—C21—C22	122.2 (3)
C6—C5—C8	121.9 (4)	N2—C22—C23	120.9 (3)
C5—C6—C7	121.6 (3)	N2—C22—C21	117.2 (3)
C5—C6—H6A	119.2	C23—C22—C21	121.9 (3)
C7—C6—H6A	119.2	C24—C23—C22	120.4 (4)
C6—C7—C2	120.1 (3)	C24—C23—H23A	119.8
C6—C7—H7A	120.0	C22—C23—H23A	119.8
C2—C7—H7A	120.0	C25—C24—C23	118.1 (4)
C5—C8—H8A	109.5	C25—C24—H24A	120.9
C5—C8—H8B	109.5	C23—C24—H24A	120.9
H8A—C8—H8B	109.5	C24—C25—C26	119.6 (4)
C5—C8—H8C	109.5	C24—C25—H25A	120.2
H8A—C8—H8C	109.5	C26—C25—H25A	120.2
H8B—C8—H8C	109.5	N2—C26—C25	122.9 (4)
O4—C9—O3	122.8 (3)	N2—C26—H26A	118.6
O4—C9—C10	120.4 (3)	C25—C26—H26A	118.6
O3—C9—C10	116.8 (3)		
